# High Efficiency CVD Graphene-lead (Pb) Cooper Pair Splitter

**DOI:** 10.1038/srep23051

**Published:** 2016-03-14

**Authors:** I. V. Borzenets, Y. Shimazaki, G. F. Jones, M. F. Craciun, S. Russo, M. Yamamoto, S. Tarucha

**Affiliations:** 1 Department of Applied Physics, University of Tokyo, Bunkyo-ku, Tokyo 113-8656, Japan; 2 Centre for Graphene Science, University of Exeter, Exeter EX4 4QL, UK; 3 PRESTO, JST, Kawaguchi-shi, Saitama 332-0012, Japan; 4 Center for Emergent Matter Science (CEMS), RIKEN, Wako-shi, Saitama 351-0198, Japan

## Abstract

Generation and manipulation of quantum entangled electrons is an important concept in quantum mechanics, and necessary for advances in quantum information processing; but not yet established in solid state systems. A promising device is a superconductor-two quantum dots Cooper pair splitter. Early nanowire based devices, while efficient, are limited in scalability and further electron manipulation. We demonstrate an optimized, high efficiency, CVD grown graphene-based Cooper pair splitter. Our device is designed to induce superconductivity in graphene via the proximity effect, resulting in both a large superconducting gap Δ = 0.5 meV, and coherence length *ξ* = 200 nm. The flat nature of the device lowers parasitic capacitance, increasing charging energy *E*_*C*_. Our design also eases geometric restrictions and minimizes output channel separation. As a result we measure a visibility of up to 86% and a splitting efficiency of up to 62%. This will pave the way towards near unity efficiencies, long distance splitting, and post-splitting electron manipulation.

A source of quantum entangled particles is essential to quantum information processing^1^,^2^. The generation of entangled photons has been achieved quite a long time ago^3^. However, the demonstration of a solid state entangler device able to source reproducibly entangled pairs of electrons in an electronic circuit on a chip has only recently been achieved^4^,^5^,^6^. According to BCS theory, electrons in a superconductor naturally form entangled spin singlets known as Cooper pairs^7^,^8^. A device that can spatially separate the entangled electrons in a superconductor into two normal leads is known as a Cooper pair splitter^9^,^10^. High efficiency Cooper pair splitting (CPS) devices have been made using the superconductor-two quantum dot design^10^,^11^. Such devices have been made using one dimensional nanowires or nanotubes with the central superconductor of Al in a T-shape^11^,^12^,^13^,^14^,^15^, with reported efficiencies of up to 90%^14^. Never the less, the one dimensional nature of the nanowire design limits further complexity of such devices.

Graphene is a single layer, crystalline sheet of carbon with hexagonal structure leading to a gamut of unique electronic properties which would contribute well to a CPS device^16^,^17^,^18^. Indeed, Cooper pair splitting in graphene quantum dots (QD) coupled to a conventional-narrow superconducting wire has recently been demonstrated^19^. In this T-shape design (essentially equivalent to the nanowire based devices) the spatially separated QDs are partially covered by the superconducting Al wire, resulting in a small charging energy of just 80 *μ*eV. Moreover the Cooper pairs tunnel directly from the Al wire into the quantum dot pinning the superconducting gap Δ and superconducting coherence length *ξ* to that of Al. These factors adversely affect the CPS efficiency of the device - only an efficiency up to 10% has been reported. In our work we design the sample to utilize the two dimensional and highly crystalline nature of graphene thus improving on all the relevant parameters required to achieve a high CPS efficiency. As a result, we see CPS efficiency that is up to 6 times greater than the previous work. Moreover, we predict that by using ultra-clean graphene devices of the same design, near unity efficiency can be achieved.

## Results

**Figure 1:**
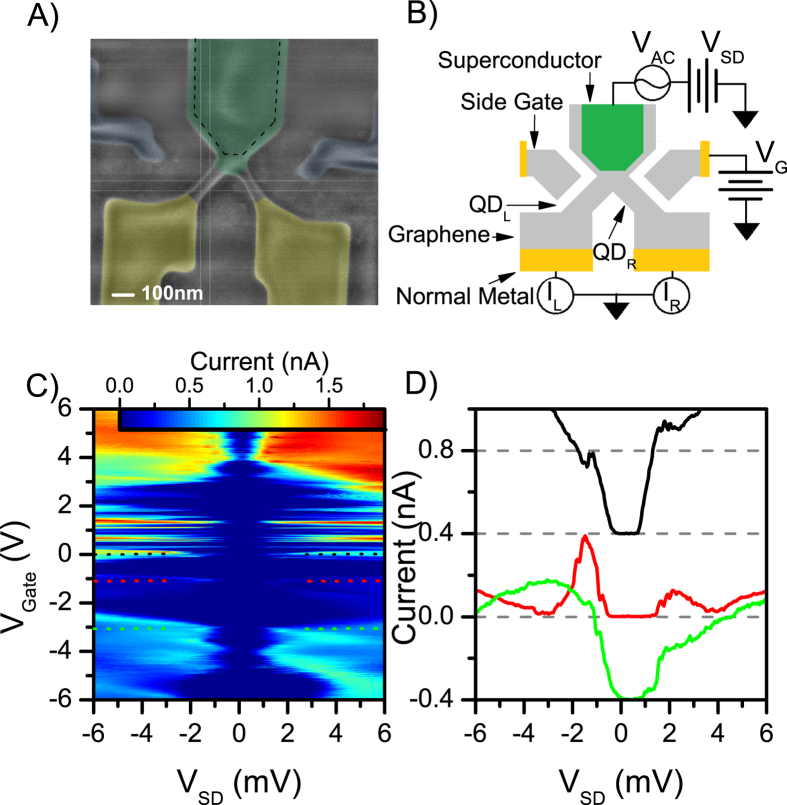
(**a**) Scanning Electron micrograph of the active area of the device prior to metal deposition. The green false color region corresponds to the central superconducting lead made from graphene. Dashed lines signify the region where lead (Pb), a conventional metal superconductor, is deposited to induce superconductivity in graphene via proximity effect. The yellow false color regions are normal regions of graphene acting as electron collectors after splitting. The superconducting and normal regions are bridged by graphene nanoribbons that form quantum dot constrictions about 70 nm width and ~150 nm length. The quantum dots are controlled by side gates: the regions tinted blue. (**b**) Device and measurement schematic. CVD graphene is patterned into a Y shape in order to minimize separation between the normal metal channels. Entrances to the Right and Left normal channels are constricted into nanoribbons. The nanoribbons form quantum dots (*QD*_*R*_, *QD*_*L*_) due to edge defects. The central electrode acts as the superconducting lead. Measurements are done via the lock-in method. AC voltage *V*_*AC*_ with a possible bias *V*_*DC*_ is applied to the central lead, currents through the Right (*I*_*R*_) and Left(*I*_*L*_) channels are measured simultaneously. *QD*_*R*_ and *QD*_*L*_ are controlled in tandem via the back gate, or individually via the self-aligned side gates. (**c**) AC current versus the bias voltage *V*_*SD*_ and back gate *V*_*BG*_ through one of the channels at base temperature and zero magnetic field. Coulomb blockade peaks separated by a gap can be seen. The presence of the peak shows that quantum dots indeed form, and a measured charging energy of ~5 meV is much larger than the superconducting gap of Pb. (**d**) Current v.s. bias voltage *V*_*SD*_ taken at the apexes of three Coulomb peaks as marked in (**c**). (The data are offset by 0.4 nA). A superconducting gap is clearly present with a maximum value of 1 meV.

The ratio between the current due to Cooper pair splitting (Δ*I*) to the background current due to two electron processes (*I*_*BG*_) is 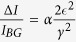
10. The prefactor *α* depends exponentially on the superconducting coherence length *ξ* and the separation of the normal-metal output channels Δ*r* according to the relation: *α* = (sin(*k*_*F*_Δ*r*)/(*k*_*F*_Δ*r*))^2^exp(−2Δ*r*/*πξ*). The variable 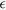 is a function of the superconducting gap Δ and the quantum dot charging energy *E*_*C*_: 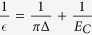. Finally, *γ* is the level resonance width, which is related to the tunneling rate, and varies with the gate voltages. We tune the design parameters to maximize *α* and 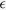. In contrast to previous works in which Cooper pairs tunnel from the superconducting metal directly into the QD, our experiments exploit three fundamental elements of novelty which increase dramatically the Cooper pair splitting efficiency. Most crucially, we induce superconductivity in bulk graphene via the proximity effect prior to splitting. Using graphene as the superconductor allows us to increase *ξ* while keeping Δ large. Moreover, the device is patterned into a true Y-shape (Fig. 1(a,b)), placing the output channels maximally close together, minimizing Δ*r*. Finally, the flat, two dimensional nature of the superconductor-quantum dot interface greatly lowers the capacitance of the quantum dots (compared to the device where the superconductor overlays the quantum dot) resulting in a large *E*_*C*_. In our Y-shape Cooper pair splitter we achieve a 100% larger value for *α* and a full order of magnitude larger 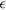 than previously demonstrated in a T-shape QD geometry with graphene^19^ (assuming that *k*_*F*_ is similar in both devices). Maximizing these variables allows us to find a gate region where the splitting visibility is up to 86% and an efficiency that is 6 times more efficient than previously demonstrated.

In a step further, we move away from the conventional method of mechanically exfoliated graphene as it has many of the same drawbacks as the nanowire based samples. Such as: the unpredictable size and location of the graphene crystals requiring that each device must be designed and aligned individually, and the small average size of the exfoliated crystals which places limits on the device complexity. Recent advances in growing macroscopic sized sheets of high quality graphene using Chemical Vapor Deposition (CVD)^20^ allow for more controlled sample design creating regular arrays of standardized devices^21^,^22^,^23^,^24^. Hence, we utilize high quality CVD graphene as the material of choice in our devices, paving the way towards integrating the Cooper pair splitter devices into more complex circuitry. Macroscopically sized sheet of Graphene grown on Copper^20^ is transferred on to a 285nm oxide *Si*/*SiO*_2_ wafer. An array of devices is defined by Cr/Au bonding pads. The active area of the graphene is defined via Electron Beam Lithography and unnecessary material is etched away using Argon Plasma. This forms the central superconducting lead, the quantum dot constrictions with self-aligned gates, and the long (much greater than the proximity effect) normal metal leads. Finally, Lead (Pb) with a sticking layer of Palladium (Pd) is used to induce superconductivity in the graphene^25^,^26^.

The sample is patterned into a Y-shaped junction to minimize separation between the normal metal channels (Fig. 1(a,b)). The distance between the outer edges of the channels at the point where they contact the central superconducting lead, Δ*r*, is 140 nm. The central lead (made from graphene) acts as the superconductor (Fig. 1(a) green hued region). Superconductivity in graphene is induced via the proximity effect by having the conventional metal superconductor lead deposited close to, but not touching the exit channels (Fig. 1(a) dashed outline). Previous works have shown that a normal metal coupled to a superconductor featured a suppressed superconducting gap Δ that decays with distance away from the superconductor^27^. The quantum dots are placed no more than 150 nm away from the conventional metal superconducting lead; while previous works have shown that graphene based jucntions of up to 1500 nm length still feature a supercurrent^25^,^26^. Quantum dot constrictions at the entrance of the normal metal leads are created by patterning graphene into regions 70 nm wide and 100–150 nm long^28^,^29^. The two quantum dot channels are individually controlled by graphene self-aligned side gate. Measurements are taken at a base temperature of 100 mK using lock-in technique. An AC voltage of (350 *μ*V) and a frequency of 447 Hz is applied to the central lead, with a possible DC bias offset. Currents through the right and the left channels are measured simultaneously. The channels can be controlled individually via the side-gates, or simultaneously via the heavily doped substrate which acts as a global back gate.

Initial characterization measurements are presented in Fig. 1(c,d). A map of the AC current through one of the channels is presented versus bias voltage and back gate. Coulomb blockade peaks separated by a zero-bias, superconducting gap can be seen. Due to the fact that quantum dots in graphene constrictions on *SiO*_2_/*Si* are formed by defects, the Coulomb peaks are irregularly spaced. However, we estimate that the quantum dot charging energy *E*_*C*_ is around 5 meV, which is greater than the energy gap of Pb (1.2 meV^8^,^30^). (Since quantum dot levels in graphene are four fold degenerate, therefore a large level spacing is not sufficient, and *E*_*C*_ ≫ Δ is required.) Having the superconducting contact in the plane of the quantum dot (as opposed to over it^19^), reduces the quantum dot capacitance, increasing *E*_*C*_. Several cuts of the current versus the bias voltage are taken at values of the back gate voltages that correspond to the apexes of the Coulomb peaks, shown in Fig. 1(d). A clear superconducting gap Δ can be seen with the energy of 0.5–1.0 meV. This value is suppressed compared to the accepted value for pure Pb. The gap feature appearing in the Coulomb blockade measurement is strong evidence that the central grapehene is indeed superconducting. Had the central lead graphene been in the normal state, it would have screened the superconducting features of the Lead (Pb) and no gap would have been observed. Direct evidence of a superconducting gap in graphene would require a scanning probe measurement^27^ and is beyond the scope of this paper. The measured value of Δ allows us to calculate a lower bound for the superconducting coherence length *ξ*. For clean graphene, the BCS superconducting coherence length is *ξ*_0_ = *ħv*_*F*_/Δ = 0.66–1.3 *μ*m, with *v*_*F*_ = 10^6^ m/s being the Fermi velocity and a maximum Δ = 0.5–1 meV^31^. In a diffusive superconductor the length is also a function of the mean free path *l* with *ξ* = (*ξ*_0_*l*)^0.5^ 32. We calculated the lower bound for the mean free path *l* = 60 nm (obtained from bulk graphene resistance), giving us a minimal coherence length of *ξ* = 199 nm. To contrast, the literature value for the superconducting coherence length of pure Lead (Pb)^33^ is *ξ*_0*Pb*_ = 83 nm: considerably less than our lead separation Δ*r*. In fact, our Pb film is not pure; the lower mean free path would suppress *ξ*_0*Pb*_ by at least a factor of 2. Using *ξ*_0*Pb*_ instead of *ξ*, but keeping all other parameters same we would see a suppression of *α* by a factor of 2 for the case of pure Pb, and more than 6 for the realistic case.

**Figure 2:**
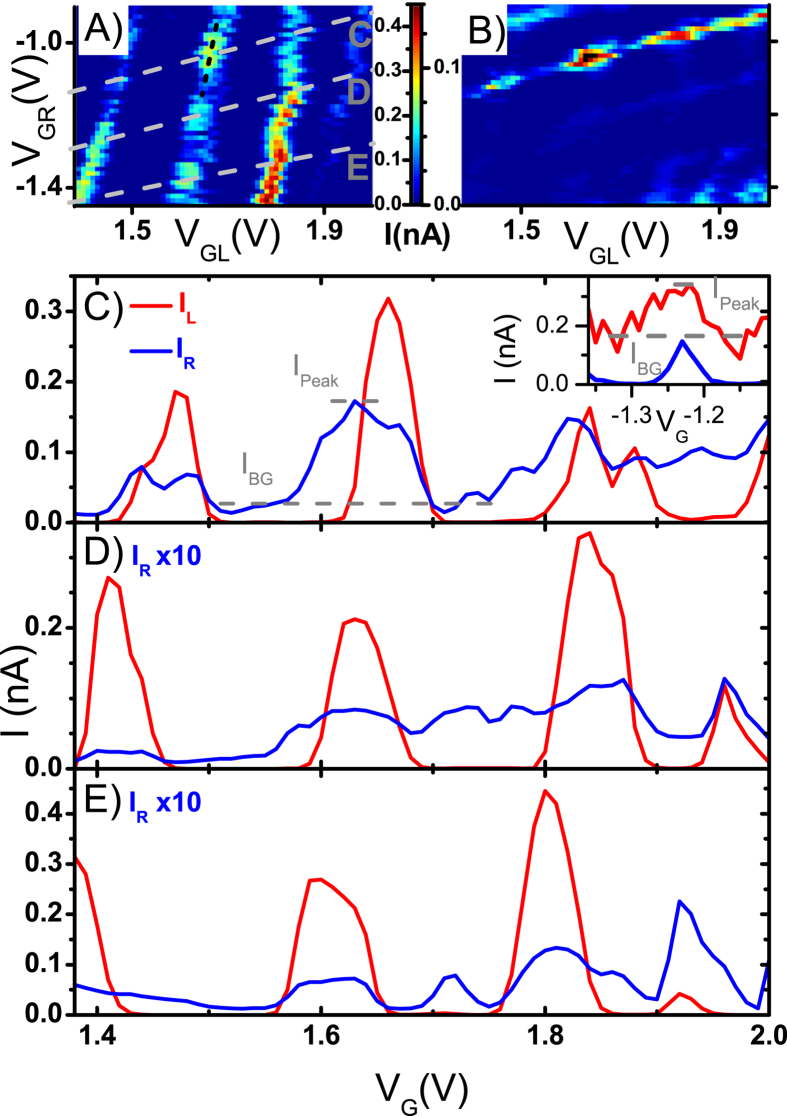
(**a,b**) Currents *I*_*L*_ (**a**) and *I*_*R*_ (**b**) versus the Left *V*_*GL*_ and Right *V*_*GR*_ side gates. Several Coulomb blockade peaks can be seen for each channel. Visually, it can be seen that the current through the channel is enhanced when both quantum dots are on resonance. (**c–e**) Current versus convolved gate voltage *V*_*G*_, *QD*_*R*_ (blue) and *QD*_*L*_ (red). The gate voltages are chosen such that *QD*_*R*_ is always kept on resonance *QD*_*L*_ is swept through several conductance peaks. Graphs C,D,E are taken for the three different resonance peaks of *QD*_*R*_ and are represented visually by the dashed lines in (**a**). Clearly, *I*_*R*_ is increased when *QD*_*L*_ goes on resonance: contrary to the classical picture, and a signature of Cooper pair splitting. C inset) Current versus convolved gate voltage *V*_*G*_, through *QD*_*R*_ (blue) and *QD*_*L*_ (red). *V*_*G*_ is chosen such that *QD*_*L*_ is kept on resonance while *QD*_*R*_ is swept through a conductance peak: represented by the black dashed line in (**a**). The background current *I*_*BG*_ and peak current *I*_*Peak*_ values used in the calculation of the splitting currents Δ*I* are denoted by the dashed lines in (**c**) for *QD*_*R*_ and (**c**) inset for *QD*_*L*_.

Grounding the back gate for stability, we now individually control the quantum dots associated with the Left (*QD*_*L*_) and Right (*QD*_*R*_) channels via the side gates. Grounding the back gate ensures that the charged carriers in bulk region of graphene are electrons as the Dirac point in unannealed graphene is consistently shifted to large negative voltages. All data from this point on are taken at zero DC bias. Figure 2(a,b) shows a map of the current *I*_*L*_ through *QD*_*L*_ (Fig. 2(a)) and current *I*_*R*_ through *QD*_*R*_ (Fig. 2(b)) versus the Left and Right side gate voltages (*V*_*GL*_, *V*_*GR*_). Several Coulomb blockade resonances can be seen for both quantum dots. The difference in efficiencies for a given quantum dot of the side gates (as well as the back gate) scales approximately with the distance of the gate to the channel constriction^34^. The cross-talk between *V*_*GL*_ and *V*_*GR*_ results in a greater variability of the quantum dot tunneling barriers increasing the tunability of *γ*.

We now look at how *I*_*R*_ evolves as *QD*_*L*_ is tuned on and off resonance^11^. For this, we look at the data with the side gate voltages tuned such that *QD*_*R*_ always remains on resonance; i.e., the data taken along the three dashed lines in Fig. 2(a) corresponding to the three resonance peaks of *QD*_*R*_. The *I*_*L*_ and *I*_*R*_ vs gate voltage *V*_*G*_ while keeping *QD*_*R*_ on resonance is presented in Fig. 2(c–e). One can see that since *QD*_*R*_ is kept on resonance, it always has a non-zero conductance. However, *QD*_*L*_ goes through several resonance peaks as the gate voltage is changed. When *QD*_*L*_ is tuned to it’s own Coulomb resonance, a clear enhancement of *I*_*R*_ can be seen. This is taken as a signature of Cooper pair splitting, as having both quantum dots on resonance allows the electrons in a Cooper pair to leave the superconductor efficiently through the left and right dot^10^,^11^. For other gate configurations CPS is suppressed since the quantum dot constrictions do not allow two-electron tunneling processes. Moreover, enhanced conductance when both channels are on resonance is contrary to a classical picture of a biased three resistor Y-junction where the current through one channel would decrease as the conductivity of the other channel increases.

For comparison, the Fig. 2(c) inset shows the change of *I*_*L*_ as *QD*_*R*_ is swept through a resonance. (The data shown in Fig. 2(c) inset is taken along the black dashed line presented in Fig. 2(a)). As with the case for *QD*_*R*_, *I*_*L*_ is enhanced when both channels are on resonance. However, *QD*_*L*_ has a much higher background current most likely due to much more open tunnel barriers of the quantum dot constriction. We analyze the Cooper pair splitting signal for the case of Coulomb resonance peak at the crossover of the dashed line C and the black dashed line in Fig. 2(a); i.e. the most prominent peak in Fig. 2(c) and the peak in the Fig. 2(c) inset. First, the background current is calculated by averaging *I*_*L*_(*I*_*R*_) when *QD*_*R*_ (*QD*_*L*_) is moved directly off-resonance. The *QD*_*R*_ background current *I*_*BGR*_ = 24.7 pA was calculated by taking the average of *I*_*R*_ for 1.51 < *V*_*G*_ < 1.56 V and 1.71 < *V*_*G*_ < 1.76 V (Fig. 2(c)). While, the *QD*_*L*_ background current *I*_*BGL*_ = 166 pA was calculated by averaging *I*_*L*_ for −1.33 < *V*_*G*_ < −1.28 V and −1.19 < *V*_*G*_ < −1.14 V (Fig. 2(c) inset). We calculate the current due to Cooper pair splitting by subtracting the background current from the peak current (the peak current is simply the highest *I*_*L*_(*I*_*R*_) for the peak studied); that is Δ*I* = *I*_*Peak*_ − *I*_*BG*_. This gives us Δ*I*_*R*_ = 0.148 nA and Δ*I*_*L*_ = 0.172 nA. Hence we find that the CPS current does not have the same value for both channels. We attribute this imbalance to the finite interdot coupling present in our device which makes it possible for the split electrons to tunnel through the graphene from the Right to the Left quantum dot^12^,^14^,^15^. In our device geometry the interdot coupling is mainly present due to the close proximity of the quantum dot entrance constrictions and the presence of a continuous graphene crystal which connects the left and the right hand side of the Y-shape devices. Moreover, *QD*_*L*_ featured much weaker tunneling barriers than *QD*_*R*_ as evidenced by the higher background current; meaning, the time the electron would spend in *QD*_*L*_ would be much shorter than in *QD*_*R*_.

We attempt to compare the measured signal levels to theoretical predictions. Unfortunately, for our case, the measurement of the Fermi wave vector *k*_*F*_ is inaccessible and our dataset is not sufficient enough to precisely extract the level resonance widths *γ*_*L*_ and *γ*_*R*_. As an estimate, the upper value for *γ* is bound by Δ while the lower bound comes from the minimum acceptable *E*_*C*_. Giving us a range 100 *μ*V ≤ *γ*_*L*_ ≤ 400 *μ*V. With the total measured ratio of splitting current to background current 
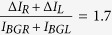
, we can obtain a range for the Fermi wave vector 1.5 × 10^5^ cm^−1^ ≤ *k*_*F*_ ≤ 2 × 10^5^ cm^−1^. The possible values of *k*_*F*_ are an order of magnitude lower than expected in normal graphene. We speculate that this discrepancy may come with the lower quasiparticle population density in superconducting graphene compared to normal graphene. (Such a cause for the lower than expected *k*_*F*_ has simliarly been postulated for the case of nanowire devices^11^).

We calculate the visibility of the Cooper pair splitting by finding the fraction of the CPS current relative to the total current: *η* = Δ*I*/(Δ*I* + *I*_*BG*_). For the resonance peak presented in Fig. 2(c) and inset we find *η*_*Right*_ = 0.86 and *η*_*Left*_ = 0.51. The lower visibility of *QD*_*L*_ is due to the high background current. Finally we calculate the splitting efficiency of our system by comparing the CPS current to the total current in the system: *s* = (Δ*I*_*R*_ + Δ*I*_*L*_)/(*I*_*Peak Left*_ + *I*_*Peak Right*_). We find the efficiency *s* = 0.62. This is significantly higher than previously reported in graphene, but much lower than needed to observe a violation of Bells inequality^35^,^36^.

**Figure 3:**
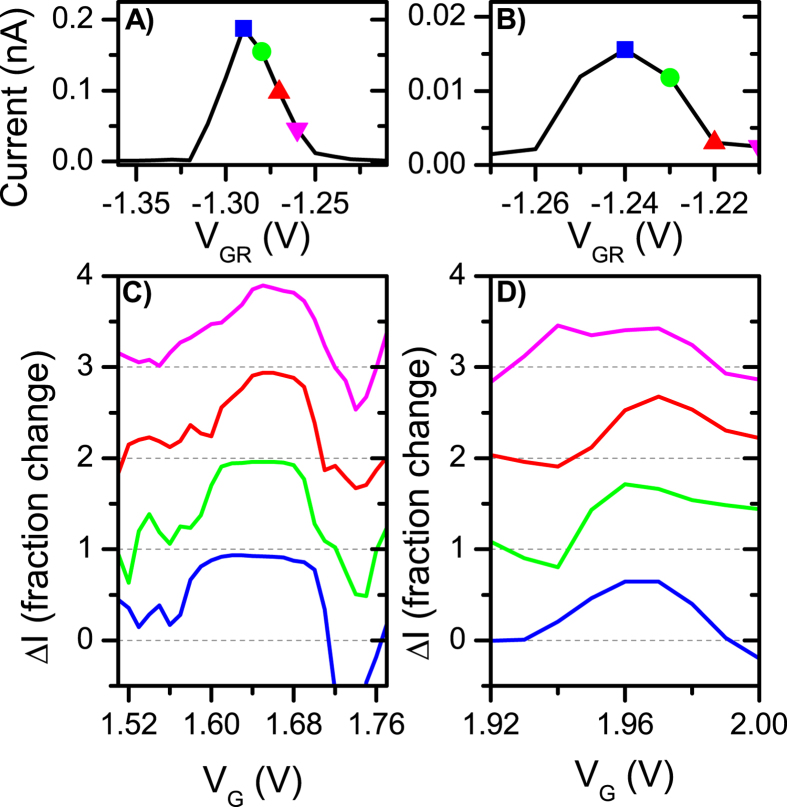
(**a,b**) *I*_*R*_ versus the Right gate *V*_*GR*_. The gate voltage region is chosen such that *QD*_*R*_ goes through a resonance peak. The peak in (**a**) corresponds to the peak presented in Fig. 2(c), while the peak in (**b**) corresponds to the peak presented in Fig. 2(d). (**c,d**) Fraction change of *I*_*R*_ versus the local background current as *QD*_*L*_ is swept through a resonance. The different curves correspond to different positions away from the resonance as presented in (**a,b**) respectively. To clarify, the green curve represents the same data as Fig. 2(c,d). All other curves represent data from curves parallel to that in Fig. 2(c,d), but more and more off resonance. The background is taken as the average of several data points immediately after *QD*_*L*_ goes off resonance. The peak current (and therefore peak Cooper pair splitting efficiency) relative to the background happens when *QD*_*R*_ is kept slightly off resonance (96% v.s.92% on resonance (**c**); and 71% v.s. 65% for (**d**)). This is in agreement with previous works. Moreover, the splitting efficiency remains high all through the resonance peak, only falling a factor of 1.6 at the edge of the resonance.

Previous works showed that the CPS efficiency was not maximum directly at the top of the quantum dot Coulomb resonance, but instead slightly off peak^10^,^14^. This was attributed to the fact that on resonance the number of electrons in a quantum dot is not well defined^10^. Therefore, we look at how the relative magnitude of the splitting signature evolves as a function of the off resonance gate shift applied to *QD*_*R*_ while sweeping the Left channel gate. Figure 3(a,b) shows cuts with respect to the Right gate (*V*_*GR*_) for resonance peaks from Fig. 2(c,d) respectively. The cuts are taken with respect to the Left gate (*V*_*GL*_) such that the absolute value of the current at the Apex is maximal. We now study how the splitting signature evolves as we sweep *QD*_*L*_ through a resonance peak while keeping *QD*_*R*_ at the positions denoted by the marks in Fig. 3a,b. The fraction increase relative to the immediate background for each location relative to the Coulomb resonance is presented in Fig. 3(c,d). To clarify, the green curves of Fig. 3(c,d) represent the same data as Fig. 2(c,d). All other curves represent data from curves parallel to that in Fig. 2(c,d), but shifted off resonance by the amount demonstrated by the points of the same color in Fig. 3(a,b). We find that the highest visibility of the CPS signature occurs for the data taken at the point immediately off resonance: 96% vs 92% at the apex for the peak in Fig. 3(a), and 71% vs 65% at the apex for peak in Fig. 3(b). In fact, the CPS signal relative to the background remains strong through the whole resonance peak, is only reduced by a factor of 1.6 for the most off-resonance point shown.

**Figure 4:**
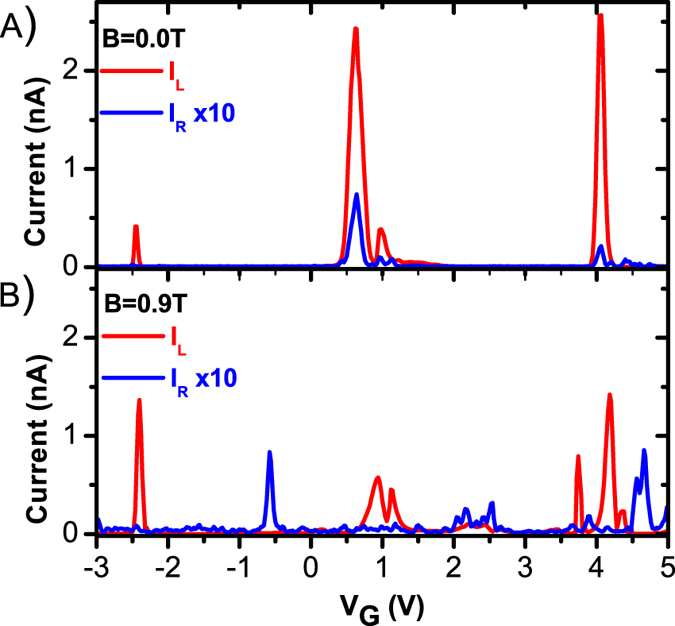
*I*_*R*_ (blue) and *I*_*L*_(red) v.s. convolved gate voltage *V*_*G*_ such that *QD*_*R*_ is kept on resonance while *QD*_*L*_ swept through several coulomb peaks. The data presented is for a device different from that in Figs 2 and 3. (**a**) Data taken at zero magnetic field. Enhancement in *I*_*R*_ can be seen when both quantum dots are on resonance. (**b**) Data taken at *B* = 0.9 T (bigger than the critical field of Pb *B*_*C*_ = 770 mT). The background current of *QD*_*R*_ is increased by close to a factor of 10 due to the disappearance of the superconducting gap. Enhancement in *I*_*R*_ no longer correlates with *QD*_*L*_ resonances demonstrating that the feature at zero field was due to the superconducting effect, supporting Cooper pair splitting. Some areas of enhanced conductance remain; we attribute this to the change in the strength of the quantum dot tunnel barriers with respect to the change in the side gate voltages.

Finally, we conclusively demonstrate that the measured non-local signal truly is due to Cooper pair splitting by verifying that the signal is only measurable when Pb is in the superconducting state. We do this by applying a perpendicular magnetic field of 0.9 T. This field is higher than the literature value for the critical field of lead (Pb) of *B*_*C*_ ~770 mT, and therefore reversibly eliminates superconductivity in our devices^37^. Presented in Fig. 4(a) is the evolution *I*_*R*_ while *QD*_*L*_ is swept through several resonances at zero magnetic field for a sample different than that in Figs 2 and 3. In this device we also confirm that *I*_*R*_ is enhanced when *QD*_*L*_ is on resonance. (The device used to collect Fig. 4 data featured more aggressive nanoribbon constrictions, thus the measured current is much lower compared to the more open device used in Figs 2 and 3.) However, when the superconducting state is broken by means of external magnetic field, no correlation between the resonances measured for *QD*_*R*_ and *QD*_*L*_ is apparent (Fig. 4(b)).

## Discussion

We achieve high efficiency Cooper pair splitting in a graphene-based, superconductor-two quantum dot junction by utilizing the unique properties of the material. The device is patterned into a true Y-shape, thus minimizing the separation between the quantum dot output channels. Previous work utilizing carbon nanotubes and demonstrating near unity efficiency, speculated that the high efficiency was aided by the nanotube region directly under the central lead (and not part of the quantum dots) becoming superconducting via proximity^14^. However, in such a device geometry characterization of the region under the central lead is experimentally inaccessible. In contrast, we explicitely induce superconductivity in the graphene via the proximity effect prior to splitting the Cooper Pairs, thus increasing the coherence length *ξ* (while maintaining large gap Δ) and ensuring that *ξ* is larger than the QD separation Δ*r*. While we do not have direct evidence that the central graphene region is superconducting, the observation of a zero-bias gap in the Coulomb blockade in strong indirect evidence. Assuming that the splitting effect is due to Lead (Pb) alone leads to a superconducting coherence length that is much smaller than the lead separation, making the observed high efficiency inaccessible. Moreover, considering that the mean free path is estimated at 60 nm, and the central graphene region prior to the quantum dots is about 150 nm long, the Cooper pairs would have decohered by the time they reached the QDs if the graphene was in the normal state. In addition, having the superconductor in plane with the quantum dots, as opposed to directly over them, we greatly increase the quantum dot charging energy *E*_*C*_. As a result, when both quantum dot channels are on resonance we see a Cooper pair splitting current that is up to 62% of the total current through the device. (Much higher than previously seen in graphene^19^). Finally, by using CVD graphene, as opposed to the more common exfoliated graphene, we eliminate the need for alignment and design tailored to individual flakes.

It is clear that in our sample design the superconducting coherence length *ξ* in graphene is suppressed due to the material being un-annealed. Simply by increasing the complexity of our device processing by adding a cleaning step or encapsulating the graphene, *ξ* can be increased by as much as an order of magnitude. This would dramatically increase the efficiency of future devices; or, alternatively achieve Cooper Pair splitting distances of more than a micron. Unlike nanowire based devices, the CVD graphene allows for more expanded complexity: moving towads measurements and manipulation of the spins of the split electron pairs, and paving a way towards applications.

## Methods

### Sample Preparation

Graphene is grown on Copper via Chemical vapor deposition method with (minimal defects) and domain sizes of ~100 *μ*m, which is much larger than the critical area of the device of a few *μ*m^20^. The CVD graphene is cut into macroscopic squares of approximately 5 mm per side and transferred on to a 297 nm oxide *Si*/*SiO*_2_ wafer using a standard *FeCl*_3_, PMMA transfer method^21^,^22^. Electron beam lithography (EBL) with PMMA as a resist and standard exposure conditions is used to define metal and etching patterns. An array of 12/150 nm Cr/Au bonding pads which includes an alignment pattern is deposited on the wafer using an electron beam evaporator. (Prior to evaporating bonding pads, the sample was etched using Ar plasma for 20 seconds at 10 W, and a gas flow of 5 sccm to remove graphene under the metal). As a second step, the active area of the graphene is defined with EBL and unnecessary material is etched away using Argon Plasma (40–60 seconds at 10 W and 5 sccm gas flow). This defines the central superconducting lead, the quantum dot constrictions with self-aligned gates, and the long (much greater than the proximity effect) normal metal leads. Finally, the superconducting contact is deposited by electron beam evaporation (Pd/Pb with 6/120 nm thickness)^25^,^26^.

### Measurement

Measurements are taken in a Oxford Instruments MX-100 dilution refrigerator at the base temperature of 100 mK using lock-in technique. The sample is isolated from the environment by a Copper can, and filtered using RC *π* filters for low frequencies, followed by Copper powder filters for high frequencies, with an overall cutoff frequency of 10 kHz. An AC voltage of (350 *μ*V) and a frequency of 447 Hz is applied to the central lead, with a possible DC bias offset. Currents through the right and the left channels are measured simultaneously via Ithaco 1211 preamplifiers. The channels can be controlled individually via the side-gates, or simultaneously via the heavily doped substrate which acts as a global back-gate.

## Additional Information

**How to cite this article**: Borzenets, I. V. *et al.* High Efficiency CVD Graphene-lead (Pb) Cooper Pair Splitter. *Sci. Rep.* **6**, 23051; doi: 10.1038/srep23051 (2016).
